# Biofouling-Resistant Impedimetric Sensor for Array High-Resolution Extracellular Potassium Monitoring in the Brain

**DOI:** 10.3390/bios6040053

**Published:** 2016-10-13

**Authors:** Ruben Machado, Nima Soltani, Suzie Dufour, Muhammad Tariqus Salam, Peter L. Carlen, Roman Genov, Michael Thompson

**Affiliations:** 1 Department of Chemistry, University of Toronto, 80 St. George Street, Toronto, ON M5S 3H6, Canada; 2 Toronto Western Research Institute, Fundamental Neurobiology Division, 60 Leonard Avenue, Toronto, ON M5T 2S8, Canada; suzie.dufour@gmail.com (S.D.); carlen@gmail.com (P.L.C.); 3 Department of Electrical and Computer Engineering, University of Toronto, 10 King’s College Road, Toronto, ON M5S 3G4, Canada; nima@eecg.toronto.edu (N.S.); tariq.hillawi@utoronto.ca (M.T.S.); roman@eecg.utoronto.ca (R.G.); 4 Institute of Biomaterials & Biomedical Engineering, University of Toronto, 164 College Street, Toronto, ON M5S 3G9, Canada

**Keywords:** biosensor, electrodes, potassium, anti-fouling, seizure, impedance

## Abstract

Extracellular potassium concentration, [K^+^]_o_, plays a fundamental role in the physiological functions of the brain. Studies investigating changes in [K^+^]_o_ have predominantly relied upon glass capillary electrodes with K^+^-sensitive solution gradients for their measurements. However, such electrodes are unsuitable for taking spatio-temporal measurements and are limited by the surface area of their tips. We illustrate seizures invoked chemically and in optogenetically modified mice using blue light exposure while impedimetrically measuring the response. A sharp decrease of 1–2 mM in [K^+^]_o_ before each spike has shown new physiological events not witnessed previously when measuring extracellular potassium concentrations during seizures in mice. We propose a novel approach that uses multichannel monolayer coated gold microelectrodes for in vivo spatio-temporal measurements of [K^+^]_o_ in a mouse brain as an improvement to the conventional glass capillary electrode.

## 1. Introduction

The extracellular fluid in the brain borders the cerebrospinal fluid (CSF), which is a clear, colorless solution that bathes the brain and spinal cord. Produced by the choroid plexus lining the ventricles of the brain, it provides nutrients for neural tissues and removes waste [1]. As such, it is critical to maintaining homeostasis throughout the nervous system. Mainly composed of water, CSF also contains 0.3% proteinaceous material, 50–80 mg/dL glucose and numerous ions such sodium and potassium [2]. In the central nervous system (CNS), glial and ependymal cells monitor and adjust the concentrations of Na^+^ and K^+^ in the fluid to facilitate normal propagation of neural impulses [3]. Regulation of these concentrations and their effects on neural function, in both states of health and disease, has been a prominent research field for a number of years. However, difficulty in the direct measurement of these ions in CSF and other biological fluids has resulted in limiting our understanding thus far.

**Figure 1:**
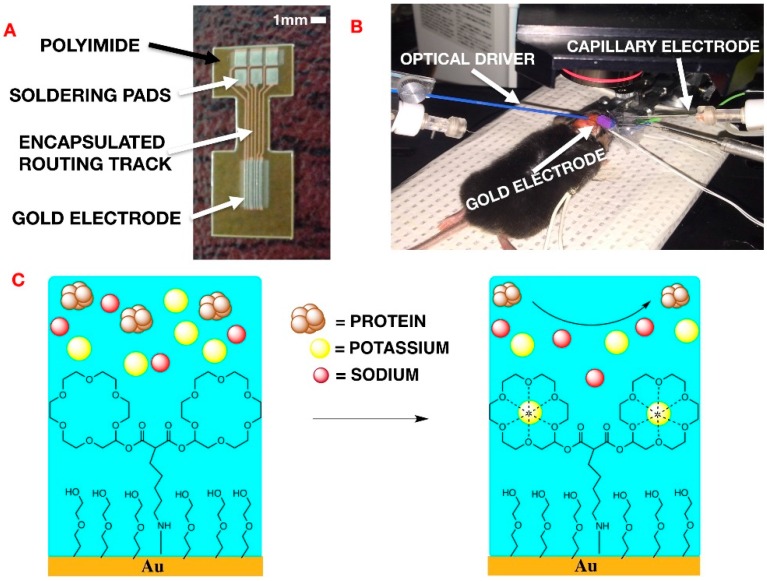
(**A**) design of multichannel gold microelectrode. It consists of a plastic film coated with gold and etched using photolithography to produce 6 pads; (**B**) experimental set-up of extracellular potassium on mouse cranium. K^+^-sensitive glass capillary electrode, reference electrode and gold-coated microelectrode were placed with 3 mm of each other in a 1 × 1 cm section of exposed cortex. Blue light stimulation was performed equidistant (1 cm) from both electrodes; and (**C**) overall 2D scheme of coated gold microelectrode. The mixed ultrathin monolayer consists of a modified 18-6-crown-ether and monoethyleneglycolthiol (MEG-SH) in 1:10 ratio, respectively.

Imbalances in [Na^+^]_o_ and [K^+^]_o_ have been implicated in numerous CNS disorders. Among these is epilepsy, which is a brain disorder that causes seizures, often with loss of consciousness [4,5]. During these episodes, abnormal neuronal and glial activity are observed in the brain. Currently, no conclusive studies have explained whether the change in [K^+^]_o_ triggers, or is the result of, seizures. For over twenty years, research into the role of K^+^ in CSF has relied upon glass capillary electrodes composed of a gradient cation-sensitive ionophore, typically a large crown-ether [6,7] with a carbon fiber conductive wire. These glass capillary electrodes contain tapered carbon fibres and use a double-barrel ion-sensitive gel system, with two gradients which specifically diffuse potassium, to measure [K^+^]_o_ [8]. The considerable shortcoming of such sensors is their lack of specificity in terms of response to the ion of interest, thereby reducing the validity of results. The use of mixed monolayer chemistry, consisting of potassium sensitive crown-ethers, allows for increased specificity in signal detection for certain cations (Figure 1C). Furthermore, capillary electrodes are limited to measuring ion concentrations at one point at a time and have an insufficient surface area for high spatial resolution. Finally, it is widely recognized that sensor surfaces are subject to protein-based fouling, which obviously leads to signal interference.

Recently, the antifouling properties of ultrathin surface-modifying coatings on gold microelectrodes have been examined [9,10]; for example, monoethyleneglycolthiol (MEG-SH) has been shown to radically reduce the amount of surface adsorption of proteins on gold surfaces [11]. This effect has been attributed to the special role played by hydration associated with the ultra-thin monolayer. Herein, we describe the addition of a modified K^+^-binding crown ether in tandem with this chemistry, which allows for the enhanced detection of this particular cation and constitutes a significantly improved substitute for the conventional ionophore-based electrode.

In terms of the particular application involved in this work, epileptic seizures are commonly studied in a mouse model. The performance of the potassium-sensitive probes was first validated in aqueous solutions containing different concentrations of cations (sodium, potassium and calcium). Additionally, to further test the potassium-sensitive probe, two independent in vivo mouse experiments were performed. Firstly, transient recording was undertaken optogenetically to evoke extracellular potassium rises in thy1:ChR2 mice (Figure 1B). The neurons of these genetically modified mice expressed a light-sensitive cation channel, which can be activated by blue light exposure. Secondly, transient [K^+^]_o_ accumulation was also recorded after accompanying seizure-like events in C57 bl6 mice. These events were initiated by the application of 1.5 M of 4-aminopyridine solution onto the exposed cortical surface as described elsewhere. For comparison purposes, extracellular [K^+^]_o_ was concurrently recorded with a glass capillary ion-sensitive recording electrode in all experiments [12,13].

## 2. Materials and Methods

### 2.1. Microelectrode Experimental Overview

A mixed ultrathin surface monolayer was added to the gold microelectrode (Figure 1A) in order to both increase device sensitivity to K^+^ in biological fluid and minimize interference of the signal caused by protein adsorption. Two 18-crown-6-ethers modified together and anchored to the gold surface using an alkyl chain, were mixed with MEG-SH in a 1:10 ratio on the gold surface. This new design was compared to the common method of capillary electrodes filled with an ion-sensitive gel. As mentioned previously [9], the performance of the potassium probe was tested in vitro as well as in an in vivo experimental context. In vivo experiments were conducted on anesthetized mice. Three electrodes were placed on an exposed cortical area, the capillary electrode, the coated gold microelectrode and the reference electrode, respectively (Figure 1B). A multi-mode optical fiber was positioned a few millimeters from the brain to allow for local light activation of light–sensitive neurons.

The chemically coated gold microelectrode pads were used to measure the extracellular [K^+^]_o_ of variation on the cortex in an in vivo mouse model. The anesthetized mouse was first probed with a 4-element gold microelectrode array (MEA) (Figure 1A) Adjacent to the microelectrode was a conventional double-barrel glass capillary electrode with a K^+^ sensitive gel placed on the exposed cerebral cortex for comparison (Figure 1B). An optogenetic fiber was also projected on the same region in the cortex to induce epileptic activity in the cerebral tissue.

The MEAs were fabricated on a 100 um thick polyimide substrate. The six electrodes were 3 mm long by 200 μm wide. The array were configured as WRWWRW (W: Working electrode, R: Reference electrode). Two of the working electrodes were selected to be wider, in case the 100 um-wide electrodes do not result in sufficient signal-noise-ratio (SNR). The electrode was wire-bonded to the soldering pads of the microelectrode array. The area of the solder pads that was not covered by epoxy was soldered to wires that connect the electrode signals to the bench top instruments. The connecting wires, which run over the probe tip and holder, interface the electrode with the function generator, the power supply, and the data acquisition terminals. The input voltage, which drives the reference pads, was sourced from the function generator (Agilent 33522B, Keysight Technologies, Santa Rosa, CA, USA). To perform impedance spectroscopy in real-time, a sinusoidal input voltage frequency was swept from 100 Hz to 1 kHz. The peak input amplitude was 4 mV, which was selected to be small enough so as not to impact the neuronal behavior of the tissue under test, but large enough to result in meaningful outputs (SNR >> 1). The sweep time was set at 100 ms based on the expected estimated bandwidth of [K^+^]_o_ variations on the cerebral cortex. The four array outputs, three working and one reference, were averaged by 10 nF capacitors immediately, and then re-digitized by a data acquisition unit (Axon Micro-Devices Digidata 1320, Molecular Devices, Sunnyvale, CA, USA).

The amplitude of the recorded current signal was more attenuated at the lower frequency end of the sweep due to the increased capacitive reactance of the electrode-CSF interface. To plot the real-time [K^+^]_o_ variation from the outputs of the four channels, the voltage and the current sweeps were processed by a system identification algorithm. The algorithm extracted the resistance (R) and capacitance (C ) parameters of the series RC model of the electrode–tissue interface.

### 2.2. Electrode Fabrication

The electrode was fabricated on a 100 μm thick polyimide sheet (Figure 1A). The polyimide substrate was cleaned with acetone and isopropyl alcohol (IPA). A thin film of polyimide (PI-2562, thickness 1.5 μm) was spin-coated (3000 revolutions per minute (RPM)) on the substrate and cured according to the PI-2562 standard recipe to provide a flat surface for improving adhesion to the following layers. Gold metallization layer (800 nm) was deposited using direct current (DC) sputtering technique and was patterned using photolithography and wet etching. The second dielectric layer (PI-2562) was spin-coated (1.5 μm) and patterned using photolithography and wet etching to create the exposed electrode and soldering pads.

### 2.3. Synthesis of Potassium Probe

#### *(**I**) bis((1,4,7,10,13,16-hexaoxacyclooctadecan*-*2-yl)methyl) malonate* *Synthesis*

To a stirred solution of 1,4,7,10,13,16-hexaoxacyclooctadecan-2-yl)methanol (0.42 mL, 1.7 mmol, 2.2 equiv.) in tetrahydrofuran (THF) (20 mL), 4-dimethylaminopyridine DMAP (20.7 mg, 0.17 mmol, 0.1 equiv.) and triethylamine (0.592 mL, 4.245 mmol, 5 equiv.) were added successfully. The mixture was cooled to 0 °C in an ice bath, where malonyl dichloride (0.082 mL, 0.849 mmol, 1 equiv) was added dropwise. The solution was allowed to heat to room temperature and run for 18 h. The resulting solution was extracted with 500 mL of ethyl acetate. Combined with organic layers was dried over anhydrous sodium sulphate, filtered, and evaporated under reduced pressure. Purification by column chromatography (hexanes/ethyl acetate: 100/0 to 85/15) finally provided malonate crown-ether II as a yellow oil (482 mg, 44% yield).

#### *1-azido*-*5-bromopentane* *Synthesis*

To a stirred solution of 1-bromo-5-chloropentane (5.49 g, 23.65 mmol, 1.1 equiv) in THF (50 mL) were successfully added sodium azide (0.9 g, 21.5 mmol, 1 equiv) at room temperature. The reaction was then refluxed for 12 h. The resulting solution was then extracted with copious amounts of diethyl ether. The combined organic layers were dried over anhydrous Na_2_SO_4_, filtered, and then evaporated under reduced pressure. Purification was achieved by Kugelrohr distillation under high vacuum to provide the desiredazide chain (0.1 mmHg, 60 °C). (2.96 g, 71% yield)

#### *(**II**) bis((1,4,7,10,13,16-hexaoxacyclooctadecan-2-yl)methyl) 2*-*(5-azidopentyl)malonate* *Synthesis*

To a stirred solution of bis((1,4,7,10,13,16-hexaoxacyclooctadecan-2-yl)methyl (482 mg, 0.735 mmol, 1 equiv.) malonate in THF (35 mL) were successfully added sodium hydride (18 mg, 0.735 mmol, 1 equiv.) at 0 °C using an ice bath. The solution was heated to room temperature where 1-azido-5-bromopentane (108 mg, 0.735 mmol, 1 equiv.) and sodium iodide (110 mg, 0.735 mmol, 1 equiv) were also added to the mixture. The mixture was stirred overnight and extracted with copious amounts of diethyl ether. The combined organic layers were dried over anhydrous Na_2_SO_4_, filtered, and then evaporated under reduced pressure. The dried extracts were then reduced with triphenylphosphine (65 mg, 0.735 mmol) in water and transferred to organic phase using hydrochloric acid in diethyl ether. Purification by column chromatography (hexanes/ethyl acetate: 100/0 to 90/10) finally provided the amine alkylated malonate crown-ether III as a pale yellow oil (398 mg, 78% yield).

^1^H NMR (CDCl3, 400 MHz) δ 6.40 (d, 2H, C-H), 4.10–4.15 (d, 4H, CH_2_), 3.40–3.50 (m, 40H, CH_2_), 1.88 (m, 2H, CH_2_), 1.35 (m, 2H, CH_2_), 1.25 (m, 2H, CH_2_), 1.20 (m, 2H, CH_2_), 1.10 (m, 2H, N-H) (299K).

### 2.4. HS-MEG-SH Synthesis

To a stirred solution of 2-(2-chloroethoxy)- ethanol (4.28 mL, 40.0 mmol, 1.0 equiv.) in MeCN (120 mL) were successively added thiourea (15.4 g, 200.0 mmol, 5.0 equiv.) and NaI (6.0 g, 40.0 mmol, 1.0 equiv.) at room temperature. The reaction was then refluxed for three days, after which volatiles were evaporated under reduced pressure. The residue was dissolved in a 1/2 (v/v) mixture of EtOH (50 mL) and H_2_O (100 mL) to which was added, carefully portionwise, powered NaOH (40.0 g, 1.0 mol, 25 equiv.) at room temperature. The reaction was then refluxed for one day. The resulting solution was carefully acidified (at 0 °C) to pH~2 by addition of concentrated (37%) HCl then repeatedly extracted with copious amounts of CH_2_Cl_2_. The combined organic layers were dried over anhydrous Na_2_SO_4_, filtered, and then evaporated under reduced pressure.

^1^H NMR (400 MHz, CDCl_3_) 3.75 (t, 2H), 3.64 (t, 2H), 3.59 (t, 2H), 2.72 (td, 2H), 2.4–2.0 (brs, 1H), 1.57 (t, 1H) (299K).

### 2.5. Gold Electrode Cleaning Procedure

Electrodes were soaked in a 1% sodium dodecyl sulfate (SDS) aqueous solution and then placed on a spinning plate for 15 min. Then, the electrodes were rinsed with tap water followed by distilled water. Next, they were washed in acetone on a spinning plate for 15 min. The electrodes were then dried under a gentle stream of nitrogen followed by nitrogen plasma-cleaned for 20 min. To avoid contamination by air pollutants, cleaned electrodes were immediately employed in the procedure of adlayer formation using freshly prepared solutions of MEG-SH surface modifier.

### 2.6. Silanization Procedure

For this experiment, 10 mL of anhydrous ethanol and 6.9 mg of HS-MEG-OH surface modifier were added into a 20 mL scintillation vial. To a second vial was added 7 mg of K^+^ Probe in 10 mL of anhydrous ethanol. Both solutions were added together in 1:10 ratios of K^+^ Probe: MEG-OH (1 mL at a time) into individual scintillation vials with a cleaned gold microelectrodes previously placed inside. Sample vials were spun on a rotating plate for 60 min. Electrodes were then rinsed (3×) with ethanol and dried under a gentle stream of nitrogen (N_2_).

#### *2-(N*-*morpholino) ethanesulfonic Acid (MES)* *Hydrate Buffer*

In addition, 28.8 mg of solid MES was dissolved in 80 mL of deionized water. pH was adjusted to approximately 5.38 via addition of NaOH pellets. The buffer was prepared within 15 min of *ethylcarbodiimide* EDC and N-hydroxysuccinimide NHS solutions.

#### *N-(3-Dimethylaminopropyl)-N′*-*ethylcarbodiimide Hydrochloride (EDC)* *Buffer*

Furthermore, 57.5 mg of EDC was dissolved in 5 mL of deionized water. The solution was stirred on a spinning plate for 5 min.

#### *N*-*Hydroxysuccinimide (NHS)* *Buffer*

In addition, 28.8 mg of NHS was dissolved in 5 mL of deionized water, followed by stirring via spinning for 5 min.

### 2.7. Coating Procedure

EDC and NHS buffers were added to 5 mL of MES buffer and portioned (in 1 mL volumes) into scintillation vials. Gold electrodes were exposed to solution and rotated for 2 h on a spinning plate (speed 4). Immediately after, electrodes were dried under a gentle stream of nitrogen (N_2_). In addition, 10 mg of K^+^ probe was dissolved in 10 mL of absolute ethanol and portioned (1 mL) into individual scintillation vials. Electrodes were placed into vials and rotated overnight on a spinning plate (speed 4), followed by drying under a gentle stream of nitrogen.

### 2.8. Confirmation of Chemical Modifications on Gold Microelectrode

**Figure 2:**
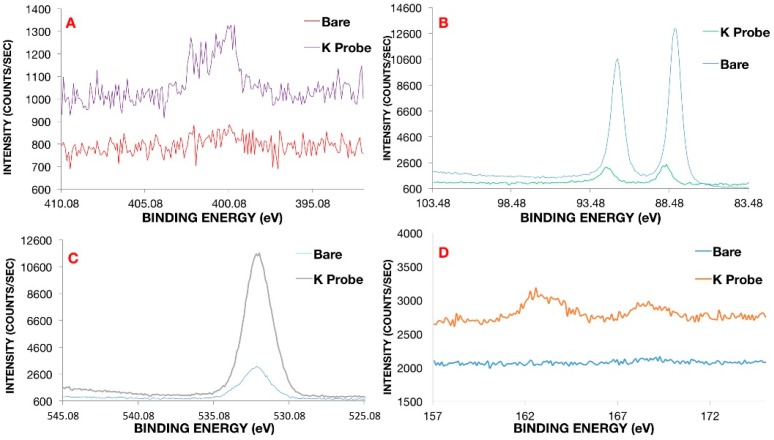
X-ray Photoelectron Spectroscopy (XPS) narrow scans on gold microelectrode for: (**A**) nitrogen; (**B**) gold; (**C**) oxygen and (**D**) sulfur, respectively. Bare cleaned gold was compared to gold coated with a mixed monolayer of K^+^ probe and MEG-SH in a 1:10 ratio.

**Figure 3:**
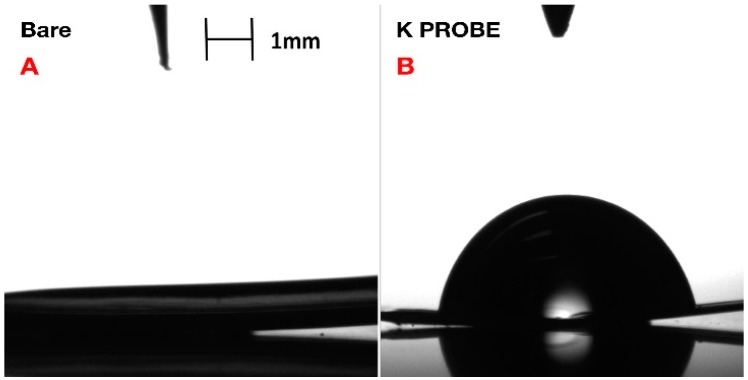
Contact Angle Geometry for (**A**) bare cleaned gold pads and (**B**) mixed monolayer coated gold pads. Heavy and light phase were measured using deionized water and air, respectively.

Confirmation of coating formation on the gold microelectrode was corroborated via X-ray photoelectron spectroscopy (XPS) (Figure 2) and contact angle-geometry (CAG) (Figure 3). CAG measurements showed consistency with previous results for MEG-SH deposited on gold [10]. As expected, bare gold displayed a higher value of contact angle due the presence of organic material on the surface (65°± 4°), while hydroxylated MEG-SH and K^+^ probe showed a significant decrease in angle due to the increased polarity associated with the hydroxyl terminated monolayer chain and oxygen rich crown ethers (14°± 5°) (Figure 3). XPS analysis was also used to verify confirmation of the K^+^ sensing coating on the gold microelectrode. Observation of the sulphur 2p signal (Figure 2D) was used to confirm the presence both monolayers, since sulphide bonds were used to attach MEG-SH and the K^+^ probe to the electrode surface. The increase in the percentage of nitrogen following K^+^ probe immobilization validated the attachment of the amine containing crown–ether probe via EDC/NHS chemistry (Figure 2A). Scans of the gold signal showed a significant decrease after coating with MEG-SH and the K^+^ probe, as expected in view of the fact that the electrode surface was covered with both carbon-based monolayers. Large increases in sulfur, carbon and oxygen amounts associated with the ether, sulfide and alkyl components of the ultrathin layer further served to demonstrate the existence of the thin film (Figure 2C,D. See also Supplementary Materials).

### 2.9. In Vivo Mouse Seizure Experimentation Protocol

In vivo experiments were conducted on adult (2–4 months old) C57 bl6 Thy1:ChR2 (Jackson Labs, Bar Harbour, ME, USA) mice. All procedures were performed following animal welfare guidelines and were approved by the Toronto Western Research Institute animal committee. Mice were initially anaesthetized with a mix of ketamine–xylaxine (respectively 95 and 5 mg/kg). A local analgesic (Sensoricaine, AstraZeneca Canada Inc., Toronto, ON, Canada) was injected subcutaneously into the area to be incised, (i.e., the area over the somatosensory cortex) after which the mice were placed in a small rodent stereotaxic frame (SG-4N Narishige, Salt Lake City, UT, USA). Prior to surgery, the level of anaesthesia was tested using the hind paw withdrawal reflex, and surgery was performed only after the disappearing of the latter. Body temperature was maintained using a thermocouple heating pad (TCAT-2DF, Physitemp, Clifton, NJ, USA). An incision was performed over the parietal bone and a 3 × 3 mm craniotomy was achieved to expose the somatosensory cortex of the right hemisphere (the craniotomy was centered 2 mm caudal to the bregma coordinate and 2 mm lateromedian). Warm saline was continuously applied over the craniotomy to prevent brain tissue damage and dehydration. A K^+^-sensitive microelectrode made of a pulled glass capillary and a glass local field potential electrode (filled with saline) were inserted into cortical layer 2–3. A reference electrode was placed in the neck muscles. The fabrication protocol of the ion-sensitive microelectrode has been described elsewhere [11], but briefly, the tip of a ≈1 MΩ silanized glass microelectrode was filled with an ion-sensitive gel (potassium ionophore I, cocktail B, Sigma-Aldrich, Mississauga, ON, Canada) and backfilled with a 0.2 M KCl solution. Electrophysiological recordings at 300 Hz were pre-amplified and sent to a high impedance amplifier (Axopatch 200b, Molecular Devices, Sunnyvale, CA, US) and digitized (Digidata 1440, Molecular Devices, Sunnyvale, CA, US) before being stored on disk. The coated gold microelectrode was carefully deposited onto the cortical surface with a micromanipulator in proximity to the glass electrodes. For optogenetic experiments, a multimode optical fiber was used to deliver blue light onto the cortex. For epilepsy experiments, a solution of 1.5 mM 4-aminopyridine was applied topically to induce recurrent seizures.

## 3. Results

### 3.1. Impedance Calibrations and Responses

**Figure 4:**
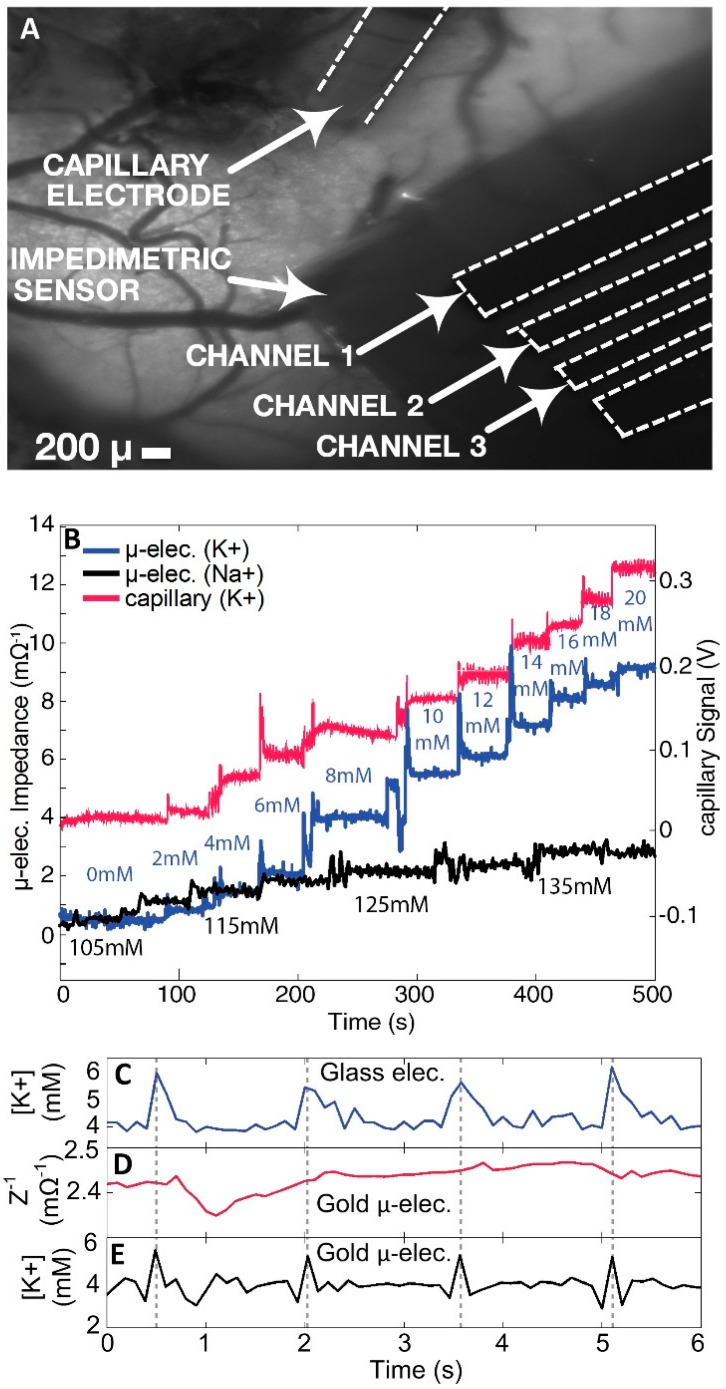
(**A**) raw fluorescence image of mouse cranial exposed window, with 10X magnification: the glass capillary electrode (**top**), and monolayer gold coated electrode (**right**); (**B**) calibration curve of both the glass capillary electrode (**red**) and coated gold microelectrode (**blue**) for varying concentrations of KCl and NaCl. Sodium concentrations were also evaluated for the gold microelectrode using NaCl in simulated CSF to determine alternative cation sensitivity. While a slight increase in signal was achieved, it was not as significant as for potassium. Simulated CSF solutions containing 2.5–22.5 mM KCl were changed in 2 mM increments. Each solution also contained: NaCl 120 mM, glucose 10 mM, NaHCO_3_ 22 mM, and 2.6 mM NaH_2_PO_4_·H_2_O, MgSO_4_ and CaCl_2_. The pH was adjusted to 7.4. The potassium-sensitive glass capillary electrode was connected to the same power supply set as the gold microelectrode, which had a built-in voltage gain to amplify the signal, resulting in heightened sensitivity to the cation concentration; (**C**–**E**) In vivo recordings of extracellular potassium during optogenetically elicited responses for both glass capillary (**blue**) and coated gold microelectrodes (**black**). Raw impedance recordings for the coated gold microelectrode were also represented for comparison (**red**). A blue light driver was pulsed every 1.5 s to initiate a potassium spike. Gold microelectrode measurements were recorded at 300 Hz.

Turning to the impedance-based electrochemistry, calibration of the electrode was achieved using simple solutions of KCl ranging from 0 to 20 mM. Increases in signal for each increase in potassium concentration were observed for both capillary and gold microelectrodes. Due to the small change in voltage from each differing concentration, amplification of signal was required to measure a response for both electrodes, resulting in stepwise rather than ratiometric increases in impedance (Figure 4B). Responses with respect to impedance measurement for each calibration point of the gold microelectrode were slightly faster in comparison with those obtained from the capillary electrode. We attribute this result to the rapid equilibration of the coated gold microelectrode with the change in concentration of [K^+^]_o_. The ionophore gradient present in the capillary electrode required a small amount of time to exchange cations and produce a change in voltage, resulting in a slight lag in the signal (Figure 4B).

**Figure 5:**
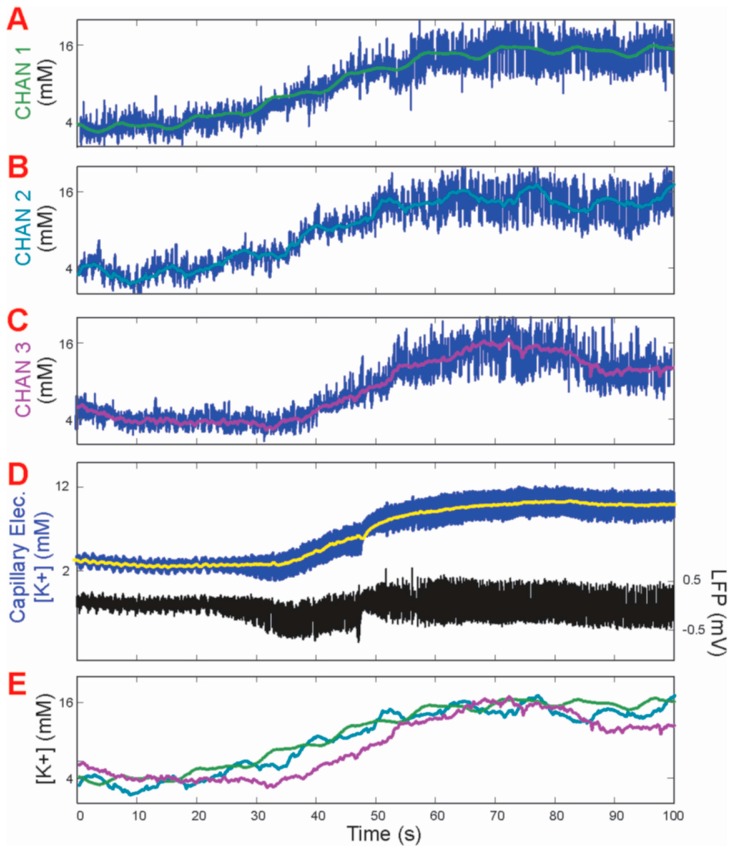
(**A**–**C**) At the onset of a 4-AP–induced focal neocortical seizure, [K^+^]_o_ measurements were shown using the coated gold microelectrodes for three individual channels as per Figure 5A–C with smoothed signals overlying the raw signal; (**D**) [K^+^]_o_ measurements for capillary electrode (**left** axis) and electroencephalogram signal from reference electrode (**right** axis); and (**E**) overlay of all three coated gold microelectrode channels with [K^+^]_o_ smoothed measurements, showing a delayed response in channel 3 which was apparently farthest from the seizure-onset zone, here demonstrating spatio-temporal specificity. Due to the plateau of the signal at each electrode, only the first 100 s of the 5 min measurement are displayed.

Optogenetic stimulation of the mouse cortex revealed concentrations of potassium that initially appeared to decrease slightly at the onset of seizure activity by approximately 1–2 mM, followed by a sharp increase of 2 mM for a period of less than 100 ms (Figure 4C). Return to equilibrium at 4 mM was immediately observed for each subsequent stimulated seizure event. When examining sensing times for optogenetic epileptic seizures, a clear distinction in peak width was observed between both capillary and gold electrodes respectively (Figure 4C). Of the two, the coated gold microelectrode exhibited reduced signal width and more consistent K^+^ peak spikes between seizure events. The difference in measuring speed between the capillary and gold microelectrode appeared to be approximately 50%, on the order of hundreds of milliseconds. The real-time [K^+^]_o_ recording by channels 1–3 of the gold microelectrode (Figure 5A–C) during and at the moment of the induced seizure showed an instantaneous rise in the extracellular [K^+^]_o_ at the seizure onset. A similar rise in potassium concentration was also recorded by a reference capillary electrode to corroborate that only potassium was being analyzed (Figure 5D).

### 3.2. Real-Time Measurement

The real-time [K^+^]_o_ variations were recorded by the glass capillary electrode which served as a reference for comparison of the extracellular potassium concentration (Figure 4D). Electrode impedance of the coated gold microelectrode was recorded simultaneously with the reference capillary electrode signal (Figure 4D,E) and real-time [K^+^]_o_ variation was extracted from the electrode capacitance using the calibration curve (Figure 4B).

It is evident from the data that the computed [K^+^]_o_ from the algorithm showed optogenetically induced [K^+^]_o_ responses that peaked precisely at the same instances as the reference glass capillary electrode. However, the initial [K^+^]_o_ decreases picked up by the coated gold microelectrodes were more rapid than those picked up by the glass capillary electrode, which were assumed to have been caused by the improved bandwidth of the MEAs as compared to the capillary electrode having a much longer time constant. The optogenetic response recorded by the coated gold microelectrode (Figure 4E) was significantly shorter than the response recorded by the capillary electrode. The shorter duration of the recorded neuronal response was attributed to the faster response rate of the proposed potassium sensitive probes on the microelectrode surface. One explanation for this phenomenon was that the high frequency voltage signal used to measure the impedance of the coated gold microelectrode has a measurement period of 1 to 100 ms, while the time constant of the capillary electrode was in the order of seconds. As depicted (Figure 4C,E), the widths between seizure spike peaks were 21%–44% smaller for the gold microelectrode than for the glass capillary electrode.

The improvement in measurement speed of the coated gold microelectrode, as compared with that of the conventional capillary probe, can be seen in both the calibration plots (Figure 4B) and the optogenetic response plots (Figure 4C–E). A reduction of nearly 50% was evident from the widths of the measured pulses. The widths of these pulses were linearly proportional to the bandwidth of the measuring probe. Thus, the improvement in response time was more difficult to discern (Figure 4C,E), as other factors, such as how and at which instant the electrode was inserted, also played a role in determining the shape and rise time of the response curve.

### 3.3. Spatial Measurements

Another improvement to the capillary electrode design was in the use of multiple points of measurement, thereby providing spatial, as well as temporal resolution. The detection of [K^+^]_o_ at only one point on the surface of the brain using the conventional glass capillary electrode represented a significant limitation. In contrast, the coated gold microelectrode used in this work, which consisted of six identical gold pads, each coated with a K^+^-sensitive mixed monolayer, were capable of sensing [K^+^]_o_ after connections for input and output have been configured. The time-averaged [K^+^]_o_ signals recorded from coated gold microelectrode channels 1–3 were reported and showed large overlap. Signals from three of the sensing pads were corroborated with point-by-point determinations performed with the glass capillary electrode. For chemically induced epileptic seizures in mice, there appeared to be similar concentrations of [K^+^]_o_ for each sensing channel of the coated gold microelectrode. A rise to approximately 10 mM for each channel was observed, although the rise of [K^+^]_o_ in channel 3 lagged behind those of the other two channels, suggesting spatio-temporal specificity (Figure 5A–C). This leads us to believe that epileptic seizures may occur in an entire region of the brain, rather than at a very specific starting point that induces the surge of extracellular K^+^ in the neighbouring regions. Another interesting observation is that the average maximal [K^+^]_o_ concentration of all three channels was maintained at ~10 mM throughout the entire seizure, with only a slight variance between neighbouring brain regions. The mechanism by which K^+^ influences epileptic seizures is still unknown. It is possible that the rise in [K^+^]_o_ caused a signaling cascade throughout the neighbouring regions of the brain.

## 4. Discussion

### 4.1. Analysis of Response Time

The peak time of each response recorded from all gold pads matched perfectly with those recorded by the reference glass capillary electrode. Additionally, very little or no degradation of the response amplitude was observed throughout the five-minute period of testing. The more rapid decay time of the [K^+^]_o_ measurement from the gold pads paralleled a well-known prolonged decay time measured from the glass capillary electrodes, albeit suggesting that the gold pad measurements more closely reflected the physiological time course of [K^+^]_o_ fluctuations.

### 4.2. Fouling Resistance of Chemical Coating

The continuation of a constant signal from the coated gold microelectrode measurements was hypothesized to be the direct result of the inclusion of the antifouling MEG-SH molecule. Research with a comparable molecule produced by silanization, rather than thiol-based attachment, has shown that such antifouling properties were strongly connected with a unique intrachain zone of hydration via internal ether oxygen atoms and distal hydroxyl moieties. The anti-fouling cation-sensing coating used in this coated gold microelectrode was composed of a 1:10 ratio of crown-ether to MEG-SH [14]. As MEG-SH has been extensively tested for antifouling activity and comprised 90% of the mixed monolayer coating (the crown-ether sharing a very similar chemical make-up of oxygen and carbon), the stable signal associated with the potassium seizure events was enough evidence to verify the previously studied anti-fouling properties of the chemical coating. Since there is no evidence of fluctuation in values throughout the seizure recording periods, it was clear that a similar hydration zone was present despite the presence of the K^+^ probe ion also mixed on the surface monolayer. In sharp contrast, bare gold surfaces have been found to be subjected to significant fouling by biological species when employed for the fabrication of devices used for in vivo measurement [15].

### 4.3. Potassium Sensitivy of Modified Crown-Ethers

Modified crown-ethers have always shown sensitivities to varying cations. Since the 18-6 crown ether has previously shown increased sensitivity to bind with potassium, their inclusion in the mixed monolayer coating was essential for specific cation measurement. While the exact mechanism of this coating has yet to be determined, we infer that as the concentration of potassium in simulated CSF increases, more potassium cations bind to the surface coating, increasing the impedance signal.

While modified crown-ethers have been shown to display particular sensitivities to characteristic cations, the unwelcome interference of other positive ions such as calcium, and especially sodium, were removed in sequence by averaging out their respective impedance at various concentrations in simulated CSF, followed by subtraction of each signal from the overall measurement. This method was also replicated for the antifouling MEG-SH monolayer since, due to a similar structure to the potassium crown ether, it also drew in numerous cations indiscriminately. Since the monolayer is composed of both structures, complete removal of interference from other cations was impossible, and the subtraction of a substantial amount of noise was performed to obtain a reliable signal for [K^+^]_o_. 

### 4.4. Analysis of Physiological Potassium Spikes

Previously, only increased spikes in extracellular potassium had been detected during seizures in mice [16]. Measurements of [K^+^]_o_ via the coated gold microelectrode displayed slight sharp decreases of approximately 2 mM before each spike in potassium concentration during seizure activity (Figure 4E). These are postulated to be physiological events happening at the cellular level, whereby the slight decrease in concentration leads to the sharp rise in [K^+^]_o_. As this has not been witnessed previously using conventional glass capillary electrodes, it appeared that the more rapid response time of the coated gold microelectrode provided new physiological information on induced epileptic seizures in mouse brains in vivo.

## 5. Conclusions

In conclusion, we have presented evidence for a much-improved K^+^ sensor for the study of epilepsy in mice through the use of mixed ultrathin surface coatings containing a K^+^-sensitive modified crown ether. The amalgamation of this novel coating with a multichannel gold electrode allowed for not only the spatial detection of extracellular potassium in CSF, but also for a reduction in non-specific adsorption of unwanted proteins, allowing for extended recording periods. An added benefit as compared to alternative methods was the increase in response time to 300 Hz for faster sensing when compared with classic glass capillary electrodes. A combination of individually specific cation-sensitive coatings for each electrode pad was one potential future aim, whereby simultaneous recordings of numerous cations with correlations to biological and seizure activities could be monitored.

## Supplementary Materials

The following are available online at www.mdpi.com/2079-6374/6/4/53/s1, Nuclear Magnetic Resonance (NMR) of final product *bis((1,4,7,10,13,16-hexaoxacyclooctadecan-2-yl)methyl) 2-(5-azidopentyl)malonate. (PDF). Impedimetric Measurements of: Potassium, Sodium and Calcium in Simulated CSF (PDF). XPS Narrow Scans of Gold Microelectrode (PDF)*.
