# Supplementary material for: Biofouling-Resistant Impedimetric Sensor for Array High-Resolution Extracellular Potassium Monitoring in the Brain
Source: Biosensors (Basel). 2016 Oct 13;6(4):53. doi: 10.3390/bios6040053 (PMC5192373; doi:10.3390/bios6040053)
Supplement: Supplementary file 1 [file biosensors-06-00053-s001.pdf]

Supplementary Materials: Bio-fouling Resistant Impedimetric Sensor Array for High-Resolution Extracellular Potassium Monitoring in the Brain

Ruben Machado, Nima Soltani, Suzie Dufour, Muhammad Tariqus Salam, Peter L. Carlen, Roman Genov and Michael Thompson

Abstract: Extracellular potassium concentration, $[K^+]_o$, plays a fundamental role in the physiological functions of the brain. Studies investigating changes in $[K+]_o$ have predominantly relied upon glass capillary electrodes with K^+-sensitive solution gradients for their measurements. However, such electrodes are unsuitable for taking spatio-temporal measurements and are limited by the small surface area of their tips. We propose a novel approach that uses multichannel gold monolayer coated microelectrodes for *in vivo* spatio-temporal measurements of $[K^+]_o$ in the mouse brain.

1. Nuclear Magnetic Resonance(NMR)

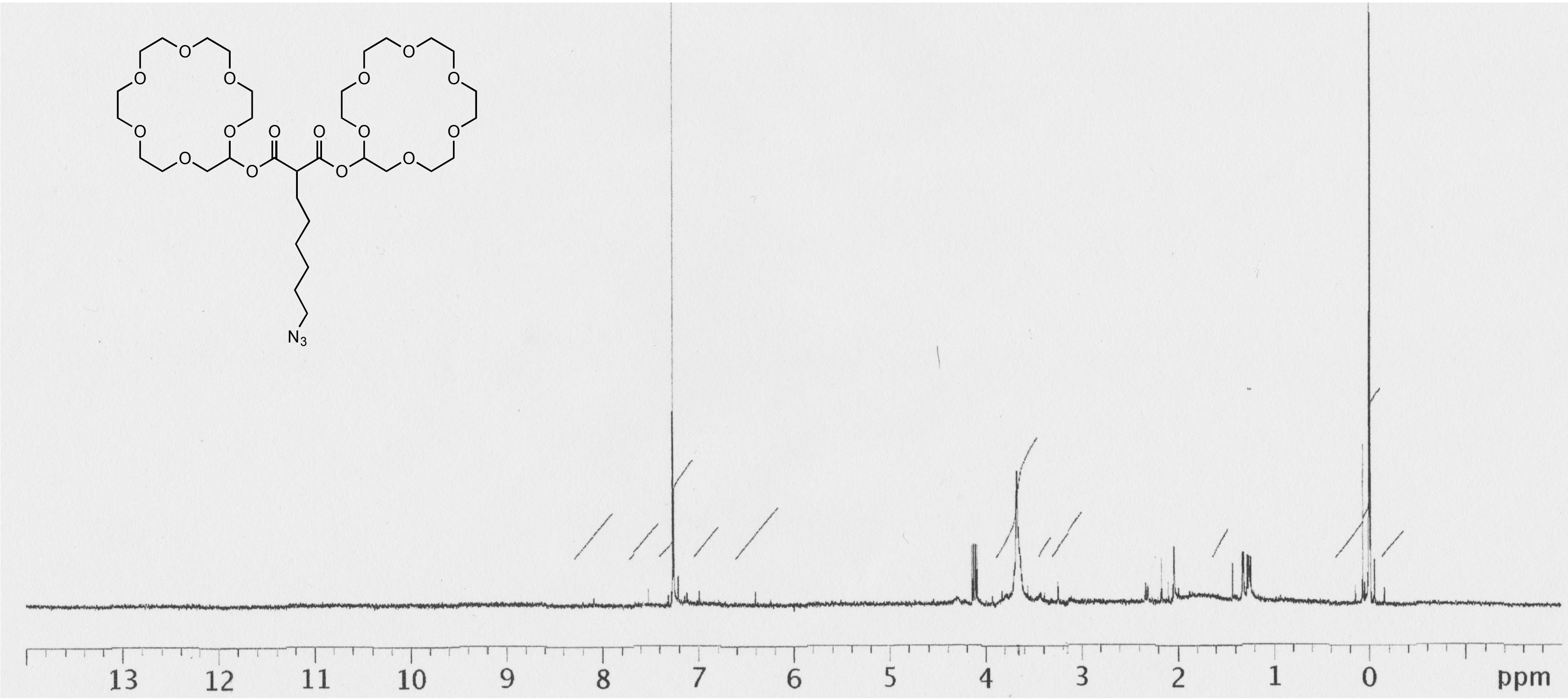

Figure S-2: NMR for ***(II)*** *bis((1,4,7,10,13,16-hexaoxacyclooctadecan-2-yl)methyl) 2-(5-azidopentyl)malonate.*

2. Impedance Measurements

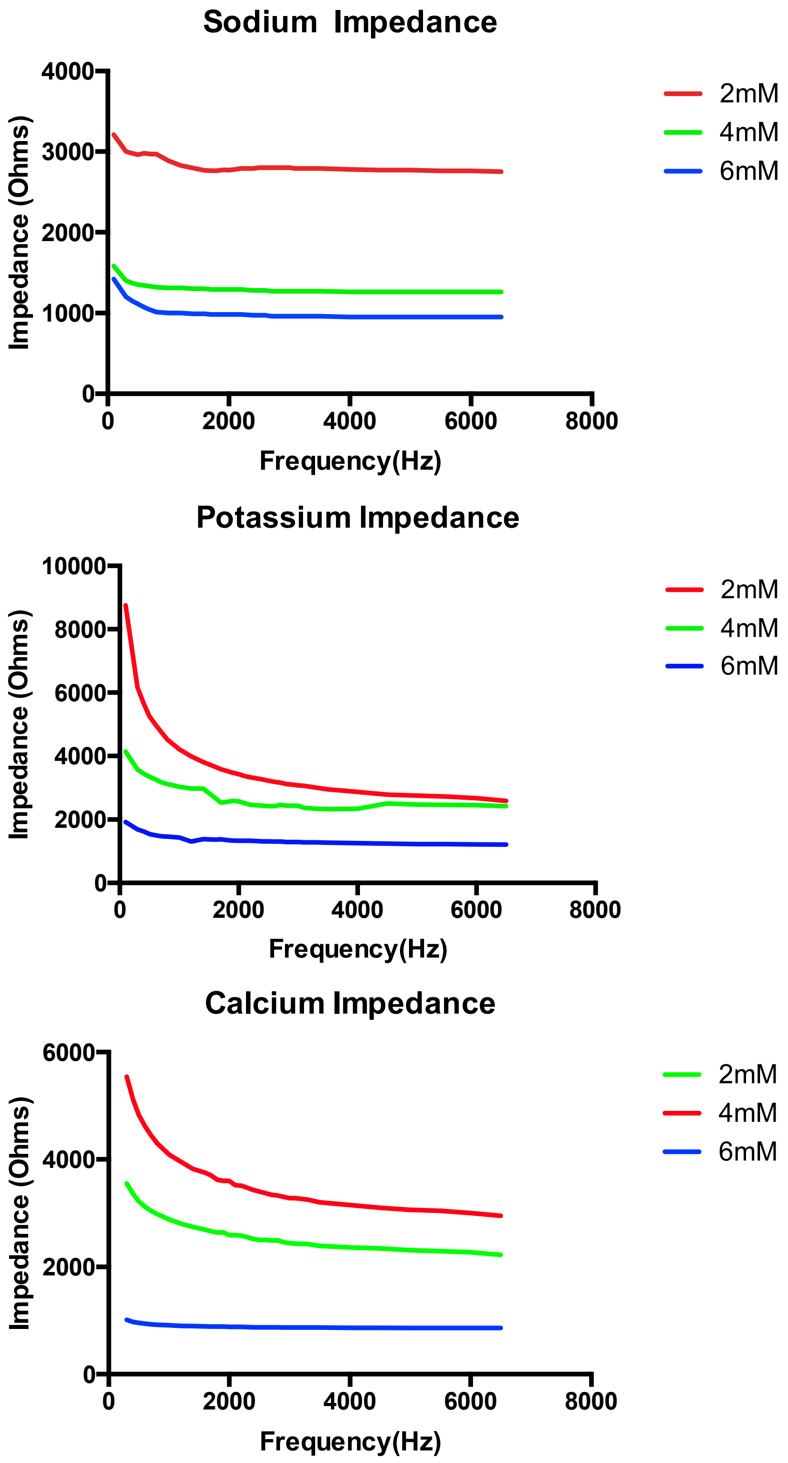

Figure S-3: Impedance measurements for: sodium(top), potassium(middle), and calcium(bottom) in simulated cerebrospinal fluid (CSF). Each solution contained: NaCl 120mM, 10 mM glucose 10mM, $NaHCO_3$ 22mM, $NaH_2PO_4{\cdot}H_2O$, $MgSO_4$ mM and $CaCl_2$ 2.6mM. Any compounds containing the cation of interest in the simulated CSF were replaced with a similar cation.

3. X-ray Photoelectron Spectroscopy (XPS)

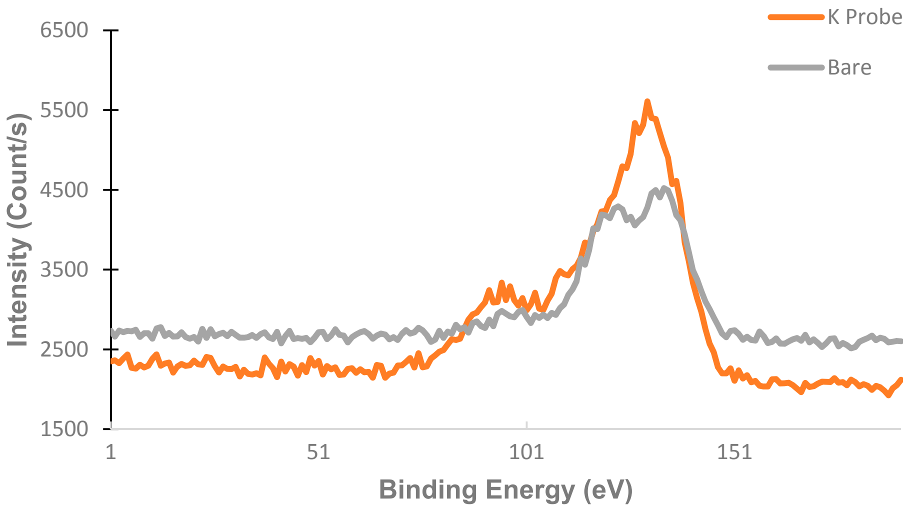

Figure S-4: X-Ray Photoelectron Spectroscopy (XPS) narrow scans for gold microelectrode for carbon. Bare cleaned gold was compared to gold coated with a mixed monolayer of K^+ probe and MEG-SH in a 1:10 ratio.
